# Intravitreal ranibizumab reduced ocular blood flow and aqueous cytokine levels and improved retinal morphology in patients with diabetic macular edema

**DOI:** 10.1038/s41598-020-78792-3

**Published:** 2020-12-10

**Authors:** Toru Mizui, Hidetaka Noma, Kanako Yasuda, Tomoe Kanemaki, Hiroshi Goto, Masahiko Shimura

**Affiliations:** 1 Department of Ophthalmology, Hachioji Medical Center, Tokyo Medical University, 1163, Tatemachi, Hachioji, Tokyo, 193-0998 Japan; 2 Department of Ophthalmology, Tokyo Medical University, Tokyo, Japan

**Keywords:** Medical research, Molecular medicine

## Abstract

We investigated the relationship between aqueous cytokine levels, changes in ocular blood flow, and morphological and functional improvements after intravitreal ranibizumab injection (IRI) in treatment-naïve eyes with center-involving diabetic macular edema (DME). Thirty-three eligible patients with DME (33 eyes) were recruited. At the first IRI, we collected a sample of aqueous humor from each eye and measured levels of the cytokines/chemokines. Mean blur rate (MBR) was used to evaluate retinal and choroidal flow by laser speckle flowgraphy at the time of the first IRI and 1 month later. One month after IRI, both retinal and choroidal MBR had significantly decreased from baseline. The reduction ratio of the retinal MBR was significantly correlated with aqueous levels of monocyte chemotactic protein (MCP)-1 and interleukin-8, and with reduction of central macular thickness, but not with improvement of best corrected visual acuity. The reduction ratio of choroidal MBR showed no statistical correlation with any cytokine levels or changes in clinical parameters. We conclude that IRI reduces both retinal and choroidal blood flow in treatment-naïve DME. Reduction of retinal blood flow correlated with regression of morphological pathology, which is regulated by the initial aqueous levels of some cytokines.

## Introduction

Diabetic macular edema (DME) is a critical complication of diabetic retinopathy, which arises from the breakdown of the blood–retinal barrier and a consequent increase in vascular permeability. It is the most frequent cause of visual impairment in the developing world^1^. Histopathologically, macular edema is an accumulation of fluid in the inner to outer plexiform layer in the retina, which may be caused by a change in retinal blood flow^2^. Elevated production of vascular endothelial growth factor (VEGF) was recently shown to be associated with DME^3^, and anti-VEGF agents, including ranibizumab and aflibercept, significantly reduce macular edema and subsequently improve visual acuity in DME^4–6^. However, the relationship between edema reduction by anti-VEGF agents and changes in retinal blood flow remains unknown.

Various ophthalmic diseases require ocular blood flow to be measured. We previously performed fluorescein angiography tracing with scanning laser ophthalmoscopy in patients with DME to investigate the relationship between perifoveal capillary blood flow velocity and macular edema^7^, but this method is invasive. Alternative noninvasive techniques include color Doppler imaging^8^, laser Doppler velocimetry^9^, and laser speckle flowgraphy (LSFG)^10–13^. Of these, LSFG is the most convenient in the clinical setting. We recently reported that the levels of several inflammatory factors in ocular fluids correlate with vascular permeability in the retina, as well as with the severity of DME^14,15^, suggesting that inflammation plays a key role in macular edema development.

In the present study, using LSFG, we evaluated retinal and choroidal blood flow before and after an intravitreal ranibizumab injection (IRI) in patients with treatment-naïve center-involving DME, and investigated the relationship between the changes in these flows and clinical parameters. Aqueous levels of cytokines at the time of IRI were also assessed to elucidate the effect of the injection on ocular blood flow.

## Results

### Demographics of patients

Between August 2015 and October 2018, 33 eyes of 33 eligible patients were included in this study. The clinical and demographic data of the patients with DME are shown in Table 1. The patients were 22 men and 11 women aged 64.8 ± 9.6 years (mean ± SD) and the mean duration of diabetes mellitus was 12.1 ± 7.7 years. The mean hemoglobin A1c level was 8.6 ± 1.9%. There were 12 patients with a history of insulin therapy (36.4%). Sixteen of the 33 patients (48.5%) had hypertension and 13 patients (39.4%) had hyperlipidemia.

**Table 1:** Clinical and demographic data of the DME patients.

Findings	DME (N = 33)
Age (years)	64.8 ± 9.6^‡^
Gender (female/male)	11/22
HbA1c (%)	8.6 ± 1.9^‡^
Duration of diabetes (years)	12.1 ± 7.7^‡^
Use of Insulin	12 (36.4%)
Hypertension	16 (48.5%)
Hyperlipidemia	13 (39.4%)
eGFR (mL/min/1.73m^2^)	63.0 ± 30.7^‡^
BUN (mg/dl)	24.5 ± 9.4^‡^
Cr (mg/dl)	1.07 ± 0.58^‡^
Baseline BCVA (logMAR)	0.46 ± 0.28^‡^
Baseline CMT (μm)	539 ± 165^‡^
MAP (mmHg)	96.1 ± 16.4^‡^
OPP (mmHg)	49.8 ± 11.1^‡^

DME = diabetic macular edema; BCVA = best-corrected visual acuity; log MAR = logarithm of the.

minimum angle of resolution; CMT = central macular thickness; MAP = mean arterial pressure;

OPP = ocular perfusion pressure; ^‡^Mean ± standard deviation (SD).

### Changes in clinical parameters

**Figure 1:**
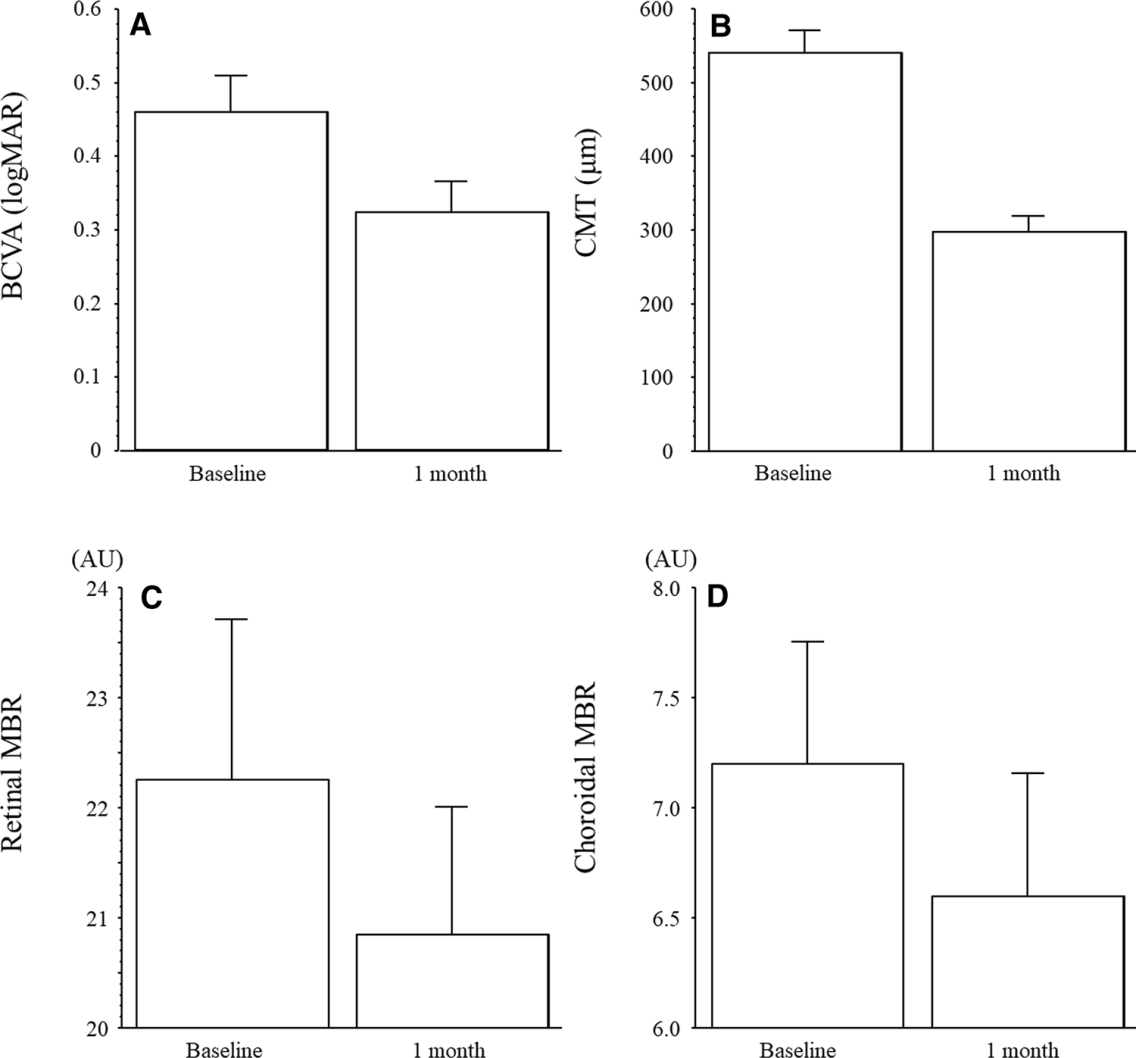
BCVA, CMT, Retinal and choroidal MBR at baseline and 1 month after IRI**.** (**A**) Significant changes were observed in mean BCVA between baseline (logMAR 0.46 ± 0.28) and 1 month (logMAR 0.32 ± 0.23; *P* < 0.001). (**B**) Significant changes were observed in mean CMT (from 539 ± 165 μm at baseline to 296 ± 117 μm at 1 month, *P* < 0.001). (**C**) Baseline retinal MBR was 22.2 ± 8.4 AU and decreased significantly 1 month (20.8 ± 6.7 AU) after IRI therapy (*P* = 0.037). (**D**) Baseline choroidal MBR was 7.20 ± 3.17 AU and decreased significantly 1 month (6.59 ± 3.19 AU) after IRI therapy (*P* = 0.003). BCVA, best corrected visual acuity; CMT, central macular thickness; MBR, mean blur rate; AU, arbitrary units; IRI, intravitreal ranibizumab injection.

Significant changes were observed in mean BCVA between baseline (logMAR 0.46 ± 0.28) and 1 month (logMAR 0.32 ± 0.23; *P* < 0.001), mean CMT (from 539 ± 165 μm at baseline to 296 ± 117 μm at 1 month, *P* < 0.001) and mean retinal MBR between baseline (22.2 ± 8.4 AU) and 1 month (20.8 ± 6.7 AU) (*P* = 0.037) (Fig. 1A–C). In addition, mean choroidal MBR decreased significantly from 7.20 ± 3.17 AU at baseline to 6.59 ± 3.19 AU at 1 month after IRI (*P* = 0.003) (Fig. 1D).

### Baseline aqueous humor cytokine levels and clinical parameters

Baseline aqueous humor levels of the 8 growth factors and cytokines tested are listed in Table 2. Baseline retinal MBR showed a significant negative correlation with levels of MCP-1 and IL-8 but not with levels of VEGF, PlGF, PDGF-AA, sICAM-1, IL-6, or IP-10 (Table 3). Baseline choroidal MBR was not significantly correlated with the levels of any growth factor or cytokine. In addition, baseline retinal MBR was not significantly correlated with any of the systemic factors (HbA1c, ρ = -0.01, *P* = 0.980; duration of diabetes, ρ = 0.11, *P* = 0.535; eGFR, ρ = -0.29, *P* = 0.097; BUN, ρ = 0.26, *P* = 0.141; and Cr, ρ = 0.09, *P* = 0.617). Baseline choroidal MBR was also not significantly correlated with any of the systemic factors (HbA1c, ρ = -0.12, *P* = 0.496; duration of diabetes, ρ = -0.03, *P* = 0.872; eGFR, ρ = -0.18, *P* = 0.310; BUN, ρ = -0.17, *P* = 0.340; and Cr, ρ = -0.26, *P* = 0.145).

**Table 2:** Aqueous humor factors/cytokines at baseline.

Factors/cytokines	Baseline
VEGF (pg/ml)	75.9 ± 74.1
PlGF (pg/ml)	9.84 ± 7.81
PDGF-AA (pg/ml)	19.5 ± 7.61
sICAM-1 (ng/ml)	30.8 ± 41.5
MCP-1 (pg/ml)	1,767 ± 926
IL-6 (pg/ml)	17.5 ± 23.5
IL-8 (pg/ml)	13.9 ± 7.60
IP-10 (pg/ml)	171 ± 232

VEGF = vascular endothelial growth factor; PlGF = placental growth factor; PDGF =

platelet-derived growth factor; MCP = monocyte chemotactic protein; sICAM = soluble.

intercellular adhesion molecule; IL = interleukin; IP-10 = interferon-inducible 10-kDa protein.

**Table 3:** Correlations between aqueous humor factors/cytokines and MBR at baseline.

Aqueous factors/cytokines	VEGF		PlGF		PDGF-AA		sICAM-1		MCP-1		IL-6		IL-8		IP-10	
Variable	*r*	*P* value	*r*	*P* value	*r*	*P* value	*r*	*P* value	*r*	*P* value	*r*	*P* value	*r*	*P* value	*r*	*P* value
Retinal MBR at Baseline	0.18	0.326	0.19	0.295	− 0.18	0.320	0.11	0.525	− 0.51	0.002	− 0.11	0.534	− 0.42	0.015	0.10	0.570
Choroidal MBR at Baseline	− 0.17	0.336	0.04	0.832	− 0.11	0.534	0.16	0.375	− 0.13	0.482	0.27	0.131	− 0.26	0.150	0.07	0.692

VEGF = vascular endothelial growth factor; PlGF = placental growth factor; PDGF = platelet-derived growth factor; sICAM = soluble intercellular adhesion molecule; MCP = monocyte chemotactic protein; IL = interleukin; *r* = correlation coefficient. Spearman’s rank-order correlation coefficients were calculated.

### Alterations in MBR and clinical parameters

**Figure 2:**
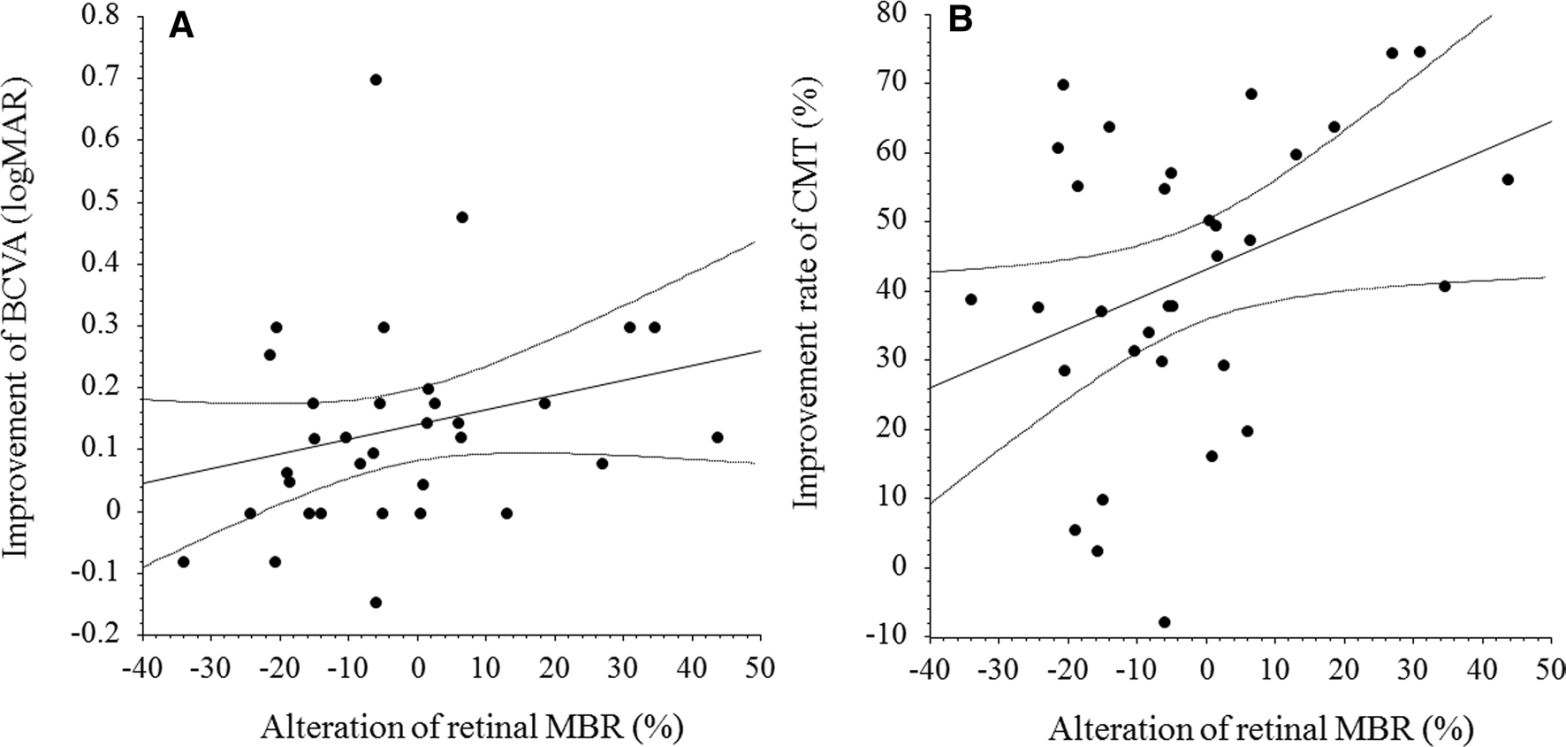
Correlation between change in retinal MBR and improvement of BCVA and CMT after IRI. (**A**) There was no significant correlation between change in retinal MBR and improvement of BCVA 1 month after IRI therapy (ρ = 0.26, *P* = 0.149). (**B**) A significant correlation was noted between change in retinal MBR and improvement of CMT at 1 month (ρ = 0.36, *P* = 0.039). BCVA, best corrected visual acuity; MBR, mean blur rate; IRI, intravitreal ranibizumab injection.

**Figure 3:**
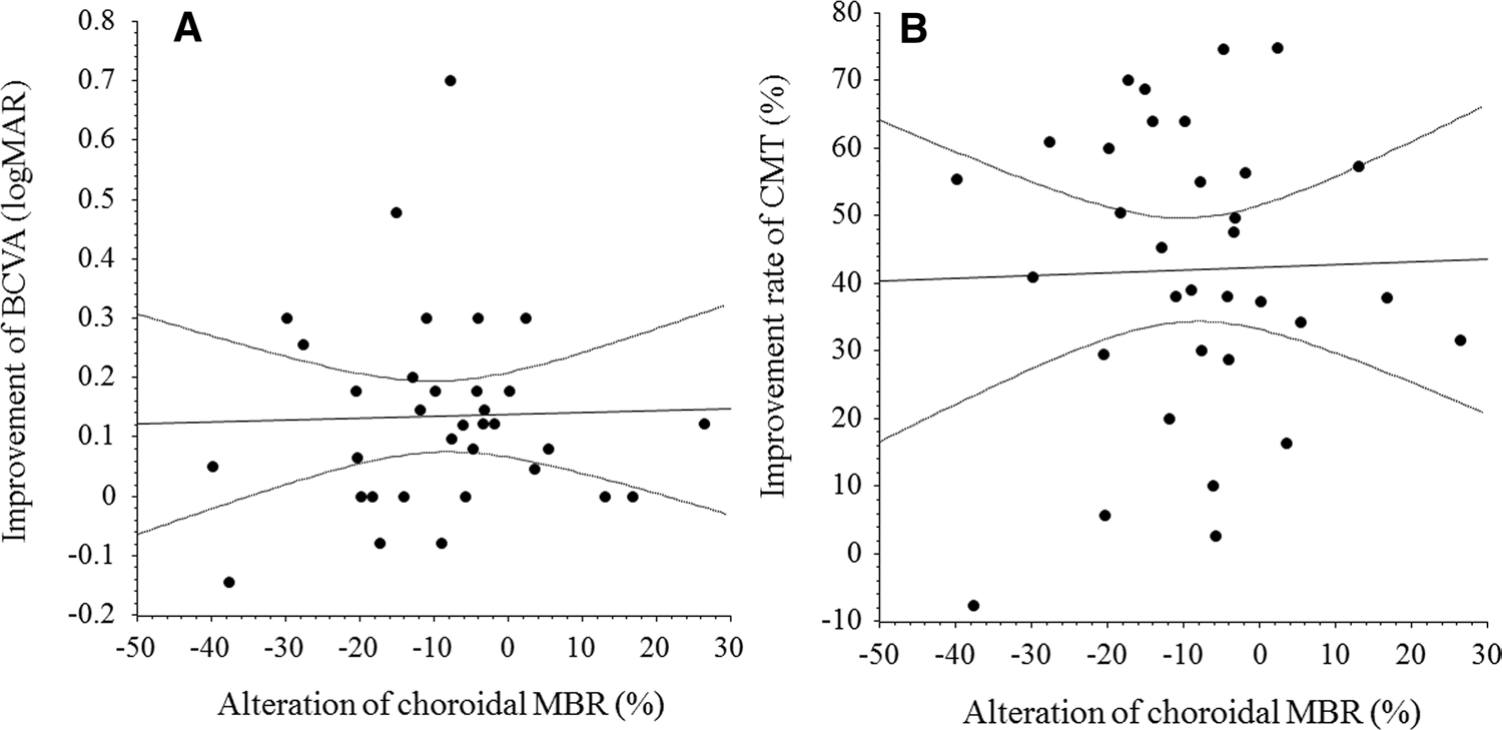
Correlation between change in choroidal MBR and improvement of BCVA or CMT after IRI. (**A**) There was no significant correlation between change in choroidal MBR and improvement of BCVA 1 month after IRI (ρ = 0.03, *P* = 0.881). (**B**) There was no significant correlation between change in choroidal MBR and improvement of CMT 1 month after IRI (ρ = 0.03, *P* = 0.884). BCVA, best corrected visual acuity; MBR, mean blur rate; CMT, central macular thickness; IRI, intravitreal ranibizumab injection.

While there was no significant correlation between change in retinal MBR and improvement in BCVA 1 month after IRI (ρ = 0.26, *P* = 0.149) (Fig. 2A), a significant correlation was noted between the change in retinal MBR and the reduction of CMT at 1 month (ρ = 0.36, *P* = 0.039) (Fig. 2B). In contrast, there were no significant correlations between change in choroidal MBR and improvement in BCVA (ρ = 0.03, *P* = 0.881) or reduction of CMT 1 month after IRI (ρ = 0.03, *P* = 0.884) (Fig. 3A and B). In addition, the change in retinal MBR was not significantly correlated with any of the systemic factors (HbA1c, ρ = -0.09, *P* = 0.604; duration of diabetes, ρ = -0.05, *P* = 0.767; eGFR, ρ = 0.02, *P* = 0.898; BUN, ρ = 0.02, *P* = 0.897; and Cr, ρ = 0.18, *P* = 0.321). The same was true for the change in choroidal MBR (HbA1c, ρ = -0.12, *P* = 0.517; duration of diabetes, ρ = -0.18, *P* = 0.313; eGFR, ρ = 0.01, *P* = 0.971; BUN, ρ = -0.09, *P* = 0.630; and Cr, ρ = -0.02, *P* = 0.903).

### Changes in MBR and aqueous cytokine levels

The change in retinal MBR showed a significant negative correlation with aqueous levels of MCP-1 and IL-8, but not VEGF, PlGF, PDGF-AA, sICAM-1, IL-6, or IP-10 (Table 4). In contrast, changes in choroidal MBR were not significantly correlated with aqueous levels of any cytokines (Table 4).

**Table 4:** Correlations between aqueous humor factors/cytokines and change in MBR 1 month after IRI.

Aqueous factors/cytokines	VEGF		PlGF		PDGF-AA		sICAM-1		MCP-1		IL-6		IL-8		IP-10	
Variable	*R*	*P* value	*R*	*P* value	*R*	*P* value	*R*	*P* value	*R*	*P* value	*R*	*P* value	*R*	*P* value	*r*	*P* value
Alteration of the retinal MBR	0.09	0.627	0.14	0.452	0.24	0.168	0.09	0.610	0.55	< 0.001	0.11	0.559	0.48	0.005	0.19	0.296
Alteration of choroidal MBR	0.12	0.511	0.01	0.940	0.31	0.072	− 0.11	0.550	0.05	0.787	0.12	0.491	0.06	0.729	− 0.27	0.126

VEGF = vascular endothelial growth factor; PlGF = placental growth factor; PDGF = platelet-derived growth factor; sICAM = soluble intercellular adhesion molecule; MCP = monocyte chemotactic protein; IL = interleukin; *r* = correlation coefficient. Spearman’s rank-order correlation coefficients were calculated.

## Discussion

This study shows that choroidal MBR significantly decreases 1 month after initiation of IRI therapy. This is consistent with a previous report by Okamoto et al.^16^ and might be because the anti-VEGF agent is vasoconstrictive in choroidal vessels^17^. We also observed a significant decrease in retinal MBR at the same time point. Ornek et al. reported that peak systolic and end diastolic velocities of the central retinal artery and nasal posterior ciliary artery decreased significantly after the injection of an anti-VEGF agent^18^. In addition, ranibizumab induces retinal arteriolar vasoconstriction^19–21^. Therefore, retinal MBR might have decreased after IRI. Couturier et al.^22^ recently reported that they detected no reperfusion of vessels or the capillary network in nonperfusion areas using two imaging techniques, ultra-widefield fluorescein angiography and swept-source widefield OCT angiography, in eyes with diabetic retinopathy after three anti-VEGF injections. Interestingly, they observed no reperfusion in nonperfusion areas, even when a reduction in dark areas was visible on fluorescein angiography. These findings and our results suggest that anti-VEGF agents do not improve retinal blood flow.

We also identified a significant negative correlation between change in retinal MBR and aqueous humor levels of MCP-1 and IL-8, whereas choroidal MBR and change in choroidal MBR were not significantly correlated with the aqueous humor levels of any factors. These findings suggest that, in patients with DME, MCP-1 and IL-8 are associated with retinal blood flow velocity but not with choroidal blood flow. MCP-1 is a chemokine that recruits monocytes/macrophages to tissues^23^. The recruitment of monocytes/macrophages to vessel walls promotes vascular permeability, potentiating DME^24^. IL-8 is a potent chemoattractant that activates neutrophils and T cells. Production of IL-8 is induced by exposure of vascular endothelial cells to hypoxia and oxidative stress^25–27^, and IL-8 promotes the adhesion of leukocytes to the vascular endothelium^28,29^. Thus, entrapment of leukocytes associated with increased rolling and adhesion of these cells by MCP-1 and IL-8 may reduce the retinal MBR in DME patients. Previously, we reported that inflammatory factors were significantly decreased after IRI^30^, so we expected that the retinal MBR would be increased after IRI, but this was not the case. In our study, only two inflammatory factors (MCP-1 and IL-8) were significantly negatively correlated with the change in retinal MBR. Ranibizumab induces retinal arteriolar vasoconstriction^19–21^, so the change in retinal MBR after IRI, rather than a change in inflammatory factors, might be responsible for vasoconstriction by the anti-VEGF agent.

Interestingly, we found a significant correlation between the change in retinal MBR and improvement of CMT 1 month after IRI therapy. This suggests that the improvement of CMT might depend on the changes in retinal blood flow. However, there was no significant positive correlation between the change in retinal MBR and improvement of BCVA. At 1 month after IRI therapy, visual acuity did not seem to have improved with CMT, suggesting that the recovery of visual acuity is a slow process and there may be a time lag between changes in CMT and improvements in BCVA. It also suggests that improvements in BCVA might not depend on changes in retinal blood flow. The change in retinal MBR could, therefore, be used as an index of CMT improvement in patients with DME who are receiving IRI therapy. Assessments of retinal blood flow using LSFG should be helpful for evaluating the effectiveness of therapeutic interventions.

This study had several limitations. First, it included a small number of patients. Second, our results are not consistent with those reported by Okamoto et al., who found that choroidal blood flow was not significantly reduced in eyes with DME^16^. The reasons for this discrepancy could be the small number patients in each study and differences in assessment criteria (for example, the study by Okamoto et al. compared a group that underwent panretinal photocoagulation with one that did not). Third, we did not measure the choroidal thickness, so the relationship between choroidal MBR and choroidal thickness remains unclear and further study is required to clarify the relationship between them in the pathogenesis of DME. Fourth, current treatment protocols for the management of patients with DME suggest that at least 3 monthly injections should be given before response is determined. However, we assessed response after a single injection because our objective was to investigate the efficacy of a single dose of an anti-VEGF agent. We plan to investigate the effects of 3 consecutive injections in a future study.

In conclusion, we found that both retinal and choroidal MBR significantly decreased one month after a single IRI. The reduction ratio of retinal MBR was significantly correlated with aqueous levels of MCP-1 and IL-8, and regression of CMT, but not with improvement in BCVA. The reduction ratio of choroidal MBR showed no statistical correlation with any cytokine levels or changes in clinical parameters. Reduction of retinal blood flow correlated with regression of morphological pathology, which is regulated by the initial aqueous levels of some cytokines. Together, these findings suggest that IRI reduces both retinal and choroidal blood flow in patients with treatment-naïve DME.

## Patients and methods

### Ethics statement

This study followed the tenets of the Declaration of Helsinki, with approval for this study obtained from the Ethics Committee of Tokyo Medical University Hachioji Medical Center (IRB No. H-131). All procedures performed in studies involving human participants were in accordance with the ethical standards of the institutional and/or national research committee and with the 1964 Helsinki declaration and its later amendments or comparable ethical standards. Informed consent was obtained from all individual participants included in the study. This study was registered in the University Hospital Medical Information Network (UMIN) clinical trials registry (UMIN000030301; 7/12/2017).

### Patients

Patients with treatment-naïve center-involving DME, of which central macular thickness (CMT) > 300 μm and best-corrected visual acuity (BCVA) < 25/30 were eligible in this study. After obtaining informed consent from each eligible patient, intravitreal ranibizumab injection (IRI; Lucentis; Novartis, Buläch, Switzerland) was administered at a dose of 0.5 mg in 0.05 ml. At the time of IRI, aqueous humor was collected to assess the concentration of cytokines/growth factors. Before and 1 month after the IRI, best corrected visual acuity (BCVA) by decimal chart and central macular thickness (CMT) by spectral-domain optical coherence tomography (OCT: Heidelberg Engineering, Heidelberg, Germany) were recorded. To facilitate data analysis, decimal BCVA data were converted to the logarithm of the minimum angle of resolution (LogMAR) values, as appropriate. At the same time, ocular blood flow was evaluated as the mean blur rate (MBR) at optic disc and fovea by LSFG^12–15,31^. All blood flow measurements were performed before intravitreal injection or anterior chamber paracentesis.

### Exclusion criteria

Patients with a history of glaucoma, uveitis, retinal diseases other than DME, rubeosis iridis, ocular infection, laser photocoagulation, intraocular surgery (including cataract surgery), and conditions causing difficulty with measurement of ocular examinations (cataract with severe opacity, vitreous hemorrhage, inadequate mydriasis, or corneal opacity), were excluded.

### Measurement of cytokines and growth factors

Under topical anesthesia, a mean volume of 0.1 mL of aqueous humor was collected by anterior chamber limbal paracentesis with a 30-gauge needle attached to an insulin syringe. Then intravitreal ranibizumab was administered through the pars plana at 3.5 mm from the limbus. Antibiotic ointment was applied for 7 days after the procedure. Immediately after collection, the aqueous humor samples were transferred to sterile plastic tubes and stored at -80 °C until analysis. As previously reported, the aqueous humor levels of cytokines (VEGF, placental growth factor (PlGF), platelet-derived growth factor (PDGF)-AA, soluble intercellular adhesion molecule-1 (sICAM-1), monocyte chemoattractant protein 1 (MCP-1), interleukin (IL)-6, IL-8, and interferon-inducible 10-kDa protein (IP-10) were measured with enzyme-linked immunosorbent assays (xMAP; Luminex Corp. Austin, TX) according to the manufacturer’s instructions (Funakoshi Corporation Ltd, Tokyo, Japan)^15^. These cytokines were selected on the basis of previous studies.

### Measurement of ocular blood flow by LSFG

The MBR, obtained by LSFG (LSFG-NAVI; Softcare Co, Ltd, Fukuoka, Japan), is a quantitative index of the relative blood flow velocity, as reported in detail previously^12,31^. Using offline analysis software (LSFG Analyzer, version 3.0.47.0), we combined all images and converted them to color-coded maps with each pixel assigned a computed MBR. The MBR was expressed in arbitrary units (AU) and displayed as a 2-dimensional, color-coded map of blood flow velocity. After manually setting a circle around the optic disc or fovea using a rubber band, we investigated the MBR within this region. The MBR in the optic disc area includes choroidal blood flow; to evaluate the retinal blood flow in the major vessels (arteries and veins) without the influence of choroidal flow, we subtracted the mean MBR of the tissue area from that of the vascular area^32,33^. To evaluate choroidal flow, we measured MBR in the avascular zone of the macula. All measurements were performed in triplicate and the mean MBR value was calculated. Eye positions were recorded by performing LSFG with an auto-tracking function, making it possible to capture the same area again during subsequent examinations with high reproducibility. To evaluate changes in retinal blood flow, the percentage change in MBR (%Δ MBR) was calculated as follows: %Δ MBR = (MBR_1month_ − MBR_baseline_)/MBR_baseline_ × 100, where MBR_baseline_ and MBR_1month_ are the MBR values at baseline (i.e., at IRI) and 1 month later, respectively.

### Systemic hemodynamics

The systolic blood pressure (SBP) and diastolic blood pressure (DBP) were measured with a mercury sphygmomanometer. The mean arterial pressure (MAP) was calculated by the following equation: MAP = DBP + 1/3 (SBP − DBP). The ocular perfusion pressure (OPP) was calculated to be: OPP = 2/3 MAP − IOP. Hypertension was diagnosed if patients were receiving antihypertensive drugs or had a blood pressure ≥ 140/90 mmHg.

### Changes of clinical parameters

To assess improvement of vision, the improvement of BCVA was calculated by subtracting the value after IRI from the value before IRI. For assessment of macular edema, the percent reduction of CMT (%ΔME) was calculated: %ΔME = (ME_baseline_ − ME_1month_)/ME_baseline_ × 100 where ME_baseline_ and ME_1 month_ were the extent of macular edema before and 1 month after intravitreal ranibizumab, respectively.

### Statistical analysis

Data are presented as the mean with ± standard deviation, as the median with interquartile range, or as frequencies. The paired *t*-test was employed to compare continuous variables between baseline and 1 month after IRI. To examine relationships among the variables, Spearman’s rank-order correlation analysis or Pearson’s correlation analysis was performed, as appropriate. Statistical significance was considered at *P* < 0.05.

## References

[1] Antcliff RJ, Marshall J (1999). The pathogenesis of edema in diabetic maculopathy. Semin. Ophthalmol..

[2] Coscas G, Cunha-Vaz J, Soubrane G (2017). Macular edema: definition and basic concepts. Dev. Ophthalmol..

[3] Funatsu H (2002). Increased levels of vascular endothelial growth factor and interleukin-6 in the aqueous humor of diabetics with macular edema. Am. J. Ophthalmol..

[4] Chun DW, Heier JS, Topping TM, Duker JS, Bankert JM (2006). A pilot study of multiple intravitreal injections of ranibizumab in patients with center-involving clinically significant diabetic macular edema. Ophthalmology.

[5] Nguyen QD (2006). Vascular endothelial growth factor is a critical stimulus for diabetic macular edema. Am. J. Ophthalmol..

[6] Wells JA (2015). Aflibercept, bevacizumab, or ranibizumab for diabetic macular edema. N. Engl. J. Med..

[7] Sakata K, Funatsu H, Harino S, Noma H, Hori S (2006). Relationship between macular microcirculation and progression of diabetic macular edema. Ophthalmology.

[8] Lieb, W.E. *et al.* Color Doppler imaging of the eye and orbit Technique and normal vascular anatomy. *Arch Ophthalmol.* 109:527–531 (1991). 10.1001/archopht.1991.01080040095036 2012555

[9] Yoshida A (1983). Retinal circulatory changes after scleral buckling procedures. Am. J. Ophthalmol..

[10] Yaoeda K (2000). Measurement of microcirculation in the optic nerve head by laser speckle flowgraphy and scanning laser Doppler flowmetry. Am. J. Ophthalmol..

[11] Tamaki Y, Araie M, Hasegawa T, Nagahara M (2001). Optic nerve head circulation after intraocular pressure reduction achieved by trabeculectomy. Ophthalmology.

[12] Sugiyama T, Araie M, Riva CE, Schmetterer L, Orgul S (2010). Use of laser speckle flowgraphy in ocular blood flow research. Acta Ophthalmol..

[13] Wang L, Cull GA, Piper C, Burgoyne CF, Fortune B (2012). Anterior and posterior optic nerve head blood flow in nonhuman primate experimental glaucoma model measured by laser speckle imaging technique and microsphere method. Invest Ophthalmol. Vis. Sci..

[14] Noma H, Mimura T, Yasuda K, Shimura M (2014). Role of inflammation in diabetic macular edema. Ophthalmologica..

[15] Noma H (2017). Aqueous humor levels of soluble vascular endothelial growth factor receptor and inflammatory factors in diabetic macular edema. Ophthalmologica..

[16] Okamoto M, Yamashita M, Ogata N (2018). Effects of intravitreal injection of ranibizumab on choroidal structure and blood flow in eyes with diabetic macular edema. Graefes Arch. Clin. Exp. Ophthalmol..

[17] Mottet B (2018). Choroidal blood flow after the first intravitreal ranibizumab injection in neovascular age-related macular degeneration patients. Acta Ophthalmol..

[18] Ornek N, Inal M, Erbahceci IE, Ogurel T, Ornek K (2015). Effect of intravitreal bevacizumab on retrobulbar blood flow of patients with diabetic macular edema. Eur. J. Ophthalmol..

[19] Papadopoulou DN, Mendrinos E, Mangioris G, Donati G, Pournaras CJ (2009). Intravitreal ranibizumab may induce retinal arteriolar vasoconstriction in patients with neovascular age-related macular degeneration. Ophthalmology.

[20] Sacu S (2011). Response of retinal vessels and retrobulbar hemodynamics to intravitreal anti-VEGF treatment in eyes with branch retinal vein occlusion. Invest. Ophthalmol. Vis. Sci..

[21] Fukami M (2017). Changes in retinal microcirculation after intravitreal ranibizumab injection in eyes with macular edema secondary to branch retinal vein occlusion. Invest. Ophthalmol. Vis. Sci..

[22] Couturier A (2019). Widefield OCT-angiography and fluorescein angiography assessments of nonperfusion in diabetic retinopathy and edema treated with anti-vascular endothelial growth factor. Ophthalmology.

[23] Yoshida S, Yoshida A, Ishibashi T, Elner SG, Elner VM (2003). Role of MCP-1 and MIP-1alpha in retinal neovascularization during postischemic inflammation in a mouse model of retinal neovascularization. J. Leukoc Biol..

[24] Tesch GH (2007). Role of macrophages in complications of type 2 diabetes. Clin. Exp. Pharmacol. Physiol..

[25] Karakurum M (1994). Hypoxic induction of interleukin-8 gene expression in human endothelial cells. J. Clin. Invest..

[26] Shono T (1996). Involvement of the transcription factor NF-kappaB in tubular morphogenesis of human microvascular endothelial cells by oxidative stress. Mol. Cell Biol..

[27] Taub DD, Anver M, Oppenheim JJ, Longo DL, Murphy WJ (1996). T lymphocyte recruitment by interleukin-8 (IL-8). IL-8-induced degranulation of neutrophils releases potent chemoattractants for human T lymphocytes both in vitro and in vivo. J. Clin. Invest..

[28] Detmers PA (1990). Neutrophil-activating protein 1/interleukin 8 stimulates the binding activity of the leukocyte adhesion receptor CD11b/CD18 on human neutrophils. J. Exp. Med..

[29] Paccaud JP, Schifferli JA, Baggiolini M (1990). NAP-1/IL-8 induces up-regulation of CR1 receptors in human neutrophil leukocytes. Biochem. Biophys. Res. Commun..

[30] Shimura M, Yasuda K, Motohashi R, Kotake O, Noma H (2017). Aqueous cytokine and growth factor levels indicate response to ranibizumab for diabetic macular oedema. Br. J. Ophthalmol..

[31] Yamada Y (2015). Retinal blood flow correlates to aqueous vascular endothelial growth factor in central retinal vein occlusion. Retina..

[32] Ubuka M (2014). Changes in the blood flow of the optic nerve head induced by different concentrations of epinephrine in intravitreal infusion during vitreous surgery. Invest. Ophthalmol. Vis. Sci..

[33] Matsumoto M (2018). Retinal blood flow after intravitreal bevacizumab is a predictive factor for outcomes of macular edema associated with central retinal vein occlusion. Retina..

